# Supplementary material for: Noninvasive continuous monitoring versus intermittent oscillometric measurements for the detection of hypotension during digestive endoscopy
Source: PLoS One. 2020 Oct 2;15(10):e0240241. doi: 10.1371/journal.pone.0240241 (PMC7531858; doi:10.1371/journal.pone.0240241)
Supplement: Attachment — Submitted filename: Response to Reviewers.docx [file pone.0240241.s002.docx]

**Point-by-point responses to the editor and the reviewer**

Dear Editor, dear Reviewer

We thank you for your thankful appreciation and this careful reviewing which has enabled us to greatly improve the quality of our manuscript entitled “Noninvasive continuous monitoring versus intermittent oscillometric measurements for the detection of hypotension during digestive endoscopy ”.

At your and the reviewer’s request, we in this letter diligently address every point that you have asked us to improve. Changes in the revised version of the manuscript are highlighted in red.

**Responses to Editor**

1. Please ensure that your manuscript meets PLOS ONE's style requirements

**Answer: We have made the necessary changes to our manuscript in order to meet PlosOne Requirements**

2. Funding statement and competing interests

**Answer: We have modified funding and competing statement according to the requirements. Modified funding and competing interest statements have been included in the new cover letter.**

3. Competing interest section

**Answer: The statement has been modified as required and indicated in the new cover letter.**

4. Supporting Information

**Answer: We have modified the supporting information following your instruction.**

**Responses to Reviewer**

1. Abstract unclear

**Answer: We have revised thoroughly the abstract to clarify the message. We have also removed abbreviations as recommended. The revised abstract length is 251 words.**

2. Normality check.

**Answer: The normality of the data distribution (SAP, MAP, SV) was verified using Kolmogorov-Smirnov test (SigmaStat®). This precision has been added to the manuscript (page 8, line 159, 163).**

3. Sample size calculation

**Answer: This was an exploratory study. Prior to its completion, we had no idea of the difference of sensitivity between continuous and intermittent pressure measurements in the detection of hypotension. Therefore, we could not estimate a sample size prospectively. This was explained in the method section (page 6, line 105-107).**

4. Non-consecutive patients

**Answer: Patients could be enrolled in the study only when, both, investigators and equipment were available. Therefore, the patients included in this study were not consecutive. This precision has been added in the manuscript (page 6, line 100-102)**

5. Inter-operator variability

**Answer: All patients were anesthetized according to our institution protocol and received similar anesthetic drugs and beseline fluid infusion. In case of hypotension, the physician in charge was free to decide to reduce the propofol infusion rate or to inject a bolus of ephedrine. Most anesthesiologists choose to modify propofol and a single patient has received a bolus of ephedrine. This is indicated page 10, line 184.**

**Regarding the endoscopic management, the inter-operator variability was mainly related to the amount of gas insufflated. Since it was not possible to quantify this parameter, we choose to collect the estimation of the endoscopist regarding his subjective appreciation of the amount of gas insufflated. This was the best we could achieve in order to provide information regarding this important aspect of the procedure since (as suggested by our results) it could greatly influence systemic hemodynamics (see Figure 4)**
